# Acute LPS sensitization and continuous infusion exacerbates hypoxic brain injury in a piglet model of neonatal encephalopathy

**DOI:** 10.1038/s41598-019-46488-y

**Published:** 2019-07-15

**Authors:** Kathryn A. Martinello, Christopher Meehan, Adnan Avdic-Belltheus, Ingran Lingam, Sara Ragab, Mariya Hristova, Cally J. Tann, Donald Peebles, Henrik Hagberg, Tim G. A. M. Wolfs, Nigel Klein, Ilias Tachtsidis, Xavier Golay, Boris W. Kramer, Bobbi Fleiss, Pierre Gressens, Nicola J. Robertson

**Affiliations:** 1 Institute for Women’s Health, University College London, London, United Kingdom; 2 Robinson Research Institute, University of Adelaide, Adelaide, Australia; 3 Maternal, Adolescent, Reproductive and Child Health Centre, Department of Infectious Disease Epidemiology, London School of Hygiene and Tropical Medicine, London, United Kingdom; 4 Centre of Perinatal Medicine & Health, Department of Clinical Sciences, Sahlgrenska Academy, Gothenburg University, Gothenburg, Sweden; 5 Centre for the Developing Brain, Department of Imaging Sciences and Biomedical Engineering, King’s College London, King’s Health Partners, St. Thomas’ Hospital, London, United Kingdom; 6 Department of Paediatrics, University of Maastricht, Maastricht, Netherlands; 7 Infection, Inflammation and Rheumatology, UCL Great Ormond Street Institute of Child Health, London, United Kingdom; 8 Medical Physics and Biomedical Engineering, University College London, London, United Kingdom; 9 Institute of Neurology, University College London, London, United Kingdom; 10 PROTECT, INSERM, Université Paris Diderot, Sorbonne Paris Cité, Paris, France; 11 Division of Neonatology, Sidra Medicine, Doha, Qatar

**Keywords:** Cell death in the nervous system, Experimental models of disease, Hypoxic-ischaemic encephalopathy, Acute inflammation

## Abstract

Co-existing infection/inflammation and birth asphyxia potentiate the risk of developing neonatal encephalopathy (NE) and adverse outcome. In a newborn piglet model we assessed the effect of *E. coli* lipopolysaccharide (LPS) infusion started 4 h prior to and continued for 48 h after hypoxia on brain cell death and systemic haematological changes compared to LPS and hypoxia alone. LPS sensitized hypoxia resulted in an increase in mortality and in brain cell death (TUNEL positive cells) throughout the whole brain, and in the internal capsule, periventricular white matter and sensorimotor cortex. LPS alone did not increase brain cell death at 48 h, despite evidence of neuroinflammation, including the greatest increases in microglial proliferation, reactive astrocytosis and cleavage of caspase-3. LPS exposure caused splenic hypertrophy and platelet count suppression. The combination of LPS and hypoxia resulted in the highest and most sustained systemic white cell count increase. These findings highlight the significant contribution of acute inflammation sensitization prior to an asphyxial insult on NE illness severity.

## Introduction

Intrapartum-related neonatal encephalopathy (NE) is estimated to affect 1.16 million babies per year, causing 287,000 deaths and resulting in 50.2 million disability adjusted life years^1^. NE is a clinical diagnosis encompassing a constellation of disordered neurological function in the newborn. Intra-partum related NE refers to NE attributable to complications around the time of birth^1,2^. The aetiology of NE is multifactorial; as well as sentinel hypoxic-ischemic events, antenatal and placental pathology^3^, genetic susceptibility^4^ and perinatal infection/inflammation^5^ contribute to brain injury and adverse outcomes^6^.

Antenatal inflammation is an established independent risk factor for the development of life long brain injury^7–9^. Epidemiological and observational clinical studies have associated perinatal inflammation with NE incidence and outcome. Clinical factors linked with inflammation, including prolonged rupture of membranes^10^ and maternal fever^3,11,12^ are associated with NE. Histological chorioamnionitis and funisitis are more commonly found in the placentas of term infants with NE compared with healthy term controls^12–15^. Fetal inflammation, leading to raised pro-inflammatory cytokines at birth, is associated with higher risks of cerebral palsy (CP) and adverse neurological sequelae in NE cohorts^16,17^. The combination of antenatal inflammation and clinically defined ‘birth asphyxia’ increases the risk of long term neurological impairment compared with either in isolation^8,18^. A number of rodent pre-clinical studies, using infection simulants such as lipopolysaccharide (LPS), demonstrate that acute infection and inflammation before hypoxia-ischemia (HI) (termed “sensitization”) lower the injury threshold and exacerbate brain injury^19–25^.

Previous studies in our newborn piglet model have used a HI insult without prior infection or inflammation^26–28^. Our aim for this study was to develop and characterize a novel large-animal model of inflammation-sensitized term intrapartum-related NE. We hypothesized that the combination of LPS (commenced 4 h before index hypoxia) and hypoxia would exacerbate brain injury measured by overall TUNEL positive (TUNEL+) cells compared to either intervention alone. The overall aim of the study was to compare LPS, Hypoxia and LPS + Hypoxia groups over 52 h for: (i) physiological changes; (ii) morbidity and mortality; (iii) amplitude integrated EEG (aEEG) background activity recovery over 48 h, which is a predictor of outcome in babies with NE^29^; (iv) histological assessment of TUNEL+ cell death in 8 brain regions at 48 h after hypoxia; and (v) systemic haematological changes. Naïve and Sham groups were included for comparisons. *Escherichia* (*E. coli*) LPS, a component of the cell wall of gram-negative bacteria and a potent endotoxin, was given to simulate perinatal gram-negative infection and inflammation. We used an established piglet model that replicates neonatal intensive care monitoring and control of physiological and metabolic parameters. This model has strong similarities to newborn infants with NE in terms of the timing of the evolution of injury after HI, pattern of injury and neuropathology^30,31^.

## Results

### Baseline characteristics and physiological parameters

Twenty-six male piglets aged <36 h were studied. One additional LPS piglet was excluded due to an LPS dosing error. Physiological parameters are shown in Table 1. Mean piglet weight (1960 g; range 1650–2100 g) was similar between groups (p = 0.216). At baseline, the mean arterial pH for the Hypoxia group was high, owing to increased CO_2_ clearance. After 4 h LPS infusion, immediately prior to hypoxia, MABP was higher in the LPS + Hypoxia group than for the saline treated Hypoxia group (p = 0.01). Immediately following insult, the Hypoxia and LPS + Hypoxia group had similarly elevated lactate and BE, and reduced pH and pO_2_, as expected in response the hypoxia. All other significant intergroup differences throughout the experiment were within target physiological range.

**Table 1:** Physiological parameters.

	Sham	LPS	Hypoxia	LPS + Hypoxia		p value
	n = 3	n = 5	n = 6	n = 5*		
	Mean (SD)	Mean (SD)	Mean (SD)	Mean (SD)	n	
**Temperature (°C)**						
Baseline	38.3 (0.5)	38.7 (0.9)	38.3 (0.8)	38.4 (0.3)	5	0.82
4 h LPS	39.0 (0.1)	38.9 (0.1)	39.0 (0.3)	38.9 (0.1)	5	0.63
End of insult (t = 0)	**38.8 (0.2)**	**38.6 (0.2)**	38.5 (0.3)	**38.1 (0.3)**	4	0.013*
1–2 h	38.8 (0.2)	**38.4 (0.2)**	**39.0 (0.3)**	38.8 (0.3)	4	0.007*
2–26 h	38.6 (0.0)	38.3 (0.2)	38.5 (0.3)	38.5 (0.1)	4	0.11
26–48 h	38.5 (0.2)	38.4 (0.2)	38.4 (0.2)	38.3 (0.0)	3	0.69
**Heart rate (bpm)**						
Baseline	178 (15)	179 (17)	170 (10)	176 (19)	5	0.76
4hLPS	193 (34)	210 (18)	186 (15)	208 (8)	5	0.13
End of insult (t = 0)	189 (30)	218 (20)	209 (9)	222 (14)	4	0.13
1–2 h	187 (22)	208 (19)	217 (16)	224 (12)	4	0.07
2–26 h	178 (11)	207 (22)	201 (32)	210 (17)	4	0.35
26–48 h	162 (15)	175 (28)	166 (46)	175 (11)	3	0.93
**Blood Pressure (mmHg)**						
Baseline	52 (5)	50 (4)	**46 (6)**	**55 (4)**	5	0.035*
4 h LPS	45 (6)	50 (6)	**43 (4)**	**54 (3)**	5	0.01*
End of insult (t = 0)	40 (5)	45 (9)	50 (10)	50 (24)	4	0.72
1–2 h	39 (6)	44 (9)	45 (10)	47 (19)	4	0.83
2–26 h	46 (4)	49 (2)	46 (4)	42 (6)	4	0.14
26–48 h	50 (5)	55 (4)	52 (6)	55 (3)	3	0.53
**pH**						
Baseline	7.40 (0.02)	**7.38 (0.08)**	**7.55 (0.09)**	7.43 (0.05)	5	0.012*
4hLPS	7.49 (0.07)	7.40 (0.08)	7.49 (0.08)	7.42 (0.05)	5	0.15
End of insult (t = 0)	**7.45 (0.03)**	**7.46 (0.10)**	**7.10 (0.13)**	**7.09 (0.05)**	4	0.000*
12 h	7.47 (0.04)	7.48 (0.10)	7.47 (0.13)	7.49 (0.09)	4	0.99
24 h	7.50 (0.03)	7.41 (0.04)	7.45 (0.04)	7.27 (0.29)	4	0.17
48 h	7.45 (0.01)	7.47 (0.07)	7.42 (0.05)	7.49 (0.08)	3	0.35
**pCO**_**2**_ **(kPa)**						
Baseline	5.8 (0.2)	**6.8 (1.6)**	**4.4 (0.7)**	6.2 (1.1)	5	0.015*
4hLPS	5.0 (0.3)	6.3 (1.1)	5.4 (1.0)	5.6 (0.7)	5	0.23
End of insult (t = 0)	5.2 (0.2)	5.9 (1.6)	6.0 (1.0)	5.6 (1.2)	4	0.41
12 h	5.1 (0.4)	5.3 (1.1)	6.2 (2.6)	4.9 (0.6)	4	0.62
24 h	4.7 (0.4)	6.2 (0.4)	5.1 (1.3)	4.7 (0.3)	4	0.07
48 h	5.2 (0.6)	5.0 (0.7)	5.5 (0.9)	4.5 (0.2)	3	0.33
**pO**_**2**_ **(kPa)**						
Baseline	16.6 (9.1)	13.6 (3.6)	10.7 (1.1)	15.1 (4.9)	5	0.29
4hLPS	12.9 (2.7)	10.8 (1.7)	11.1 (1.8)	10.1 (0.9)	5	0.23
End of insult (t = 0)	**12.9 (2.7)**	12.0 (0.6)	**6.8 (3.5)**	7.4 (3.5)	4	0.032*
12 h	13.2 (0.9)	12.9 (2.5)	14.3 (5.2)	11.5 (0.7)	4	0.66
24 h	**13.7 (1.5)**	**9.0 (0.6)**	10.5 (1.9)	10.6 (3.2)	4	0.047*
48 h	13.4 (0.3)	11.9 (3.2)	12.2 (2.2)	13.0 (2.5)	3	0.85
**Base Excess (mmol/L)**						
Baseline	2.3 (2.1)	5.2 (2.2)	6.2 (2.9)	6.2 (1.9)	5	0.15
4hLPS	3.3 (3.5)	4.0 (1.4)	6.3 (1.8)	2.8 (2.2)	5	0.07
End of insult (t = 0)	**3.3 (2.9)**	**6.5 (0.7)**	**−15.3 (5.9)**	**−17 (2.9)**	4	0.000*
12 h	5.0 (2.6)	6.4 (3.5)	8.3 (1.2)	4.5 (5.8)	4	0.36
24 h	4.7 (3.5)	4.6 (2.3)	2.5 (5.9)	−7.8 (14.9)	4	0.13
48 h	3.0 (3.0)	2.8 (1.5)	2.3 (5.5)	2.5 (4.9)	3	0.99
**Lactate (mmol/L)**						
Baseline	3.6 (1.3)	4.0 (1.4)	5.6 (1.2)	3.4 (1.4)	5	0.07
4 h LPS	3.3 (0.6)	4.4 (0.7)	4.2 (1.1)	5.4 (1.6)	5	0.12
End of insult (t = 0)	**3.3 (0.9)**	**4.1 (0.8)**	**16 (2.9)**	**15.9 (0.8)**	4	0.000*
12 h	1.7 (0.3)	3.0 (1.1)	2.6 (0.7)	5.2 (3.5)	4	0.084
24 h	1.7 (0.1)	1.6 (0.3)	4.3 (3.9)	9.3 (8.0)	4	0.08
48 h	1.6 (0.3)	0.9 (0.3)	1.5 (0.8)	1.1 (0.3)	3	0.28
**Glucose (mmol/L)**						
Baseline	4.7 (1.6)	6.9 (1.2)	5.8 (1.1)	5.2 (1.9)	5	0.18
4 h LPS	7.0 (0.9)	7.0 (1.6)	8.6 (1.4)	6.5 (2.2)	5	0.36
End of insult (t = 0)	6.2 (0.6)	6.7 (1.3)	10.7 (2.9)	10.5 (3.8)	4	0.09
12 h	5.7 (0.6)	5.8 (0.9)	7.4 (1.6)	6.6 (1.3)	4	0.17
24 h	5.1 (0.9)	6.1 (0.4)	9.4 (5.6)	9.2 (3.7)	4	0.29
48 h	5.1 (1.0)	5.5 (0.3)	6.3 (1.7)	5.4 (0.8)	3	0.52
**Potassium (mmol/L)**						
Baseline	4.2 (0.6)	4.6 (0.6)	5 (0.6)	4.5 (0.6)	5	0.38
4 h LPS	5.3 (0.4)	5.0 (0.5)	5.3 (0.7)	5.1 (0.8)	5	0.87
End of insult (t = 0)	5.3 (0.3)	5.3 (0.6)	5.2 (0.8)	5.3 (0.4)	4	0.99
12 h	6.1 (0.7)	7.1 (0.7)	6.8 (0.9)	7.7 (0.6)	4	0.09
24 h	6.4 (0.4)	6.5 (1.1)	7.3 (1.5)	8.0 (1.3)	4	0.24
48 h	4.1 (0.4)	4.9 (1.1)	7.1 (1.9)	5.3 (1.5)	3	0.044*

Physiological observations and arterial blood gas results during the experiment, mean values and standard deviation (SD). The LPS + Hypoxia column lists number of animals (n) included in each analysis, which decreases over time due to mortality in this group. p values presented are from an ANOVA. All p values < 0.05, except 48 h potassium, maintained significance following a post hoc Tukey-Kramer correction for multiple comparisons (bold indicates involved values).

### Hypoxic insult

Insult severity (determined by duration of hypoxia, duration of hypotension and thus ischemia (MABP <30 mmHg), duration of isoelectric EEG and area under the curve (AUC) FiO_2_) was similar between groups (Table 2). There was a trend towards a shorter duration of EEG <5 µV (min) in the LPS + Hypoxia group compared to the Hypoxia group (p = 0.07). End of insult blood gases were similar between groups.

**Table 2:** Insult severity.

	Hypoxia	LPS + Hypoxia	p value
Duration (min)	32 (10)	24.4 (4.5)	0.14
AUC FiO_2_ (%)	468 (177)	357 (67)	0.17
Duration of EEG <5 μV (min)	27.3 (9.6)	16.4 (6.9)	0.07
Duration of MABP <30 mmHg (min)	15 (8.2)	8.8 (4.8)	0.16
**End of Insult Arterial Gas:**			
pH	7.10 (0.13)	7.09 (0.03)	0.92
pCO_2_ (kPa)	6.0 (1.0)	5.6 (1.2)	0.56
Base Excess (mmol/l)	−15.3 (5.9)	−17 (2.9)	0.62
Lactate (mmol/L)	16.0 (2.9)	15.9 (0.8)	0.93

Markers of insult severity, mean with standard deviation (SD). There was no significant difference between insult severity parameters between the Hypoxia and LPS + Hypoxia groups. AUC = area under the curve, MABP = mean arterial blood pressure.

### Survival and clinical illness severity

Three of the five LPS + Hypoxia piglets died prior to experiment completion compared with none in the other groups (p = 0.022). One piglet died within minutes of insult cessation during the resuscitation phase: this piglet’s brain was the only one not obtained for later histological analysis. Two further piglets arrested at approximately 24 h with preceding refractory hypotension. One LPS and one Hypoxia piglet suffered respiratory arrests due to mechanical airway obstruction/equipment failure. Both were promptly resuscitated and completed the full duration of the experiment.

There was no significant difference in inotrope or saline bolus requirement between groups (Table 3). T-test comparison of hypoxia exposed versus non-exposed demonstrated greater doses of dopamine, dobutamine, adrenaline and saline for hypoxia exposed piglets (p = 0.019, p = 0.046, p = 0.031 and p = 0.011 respectively), although this study was not powered to detect differences in peripheral outcome measures. LPS exposure did not effect overall inotrope requirement (p ≥ 0.42). One Sham, one LPS and two Hypoxia piglets needed no inotropes. All piglets in the LPS + Hypoxia group required inotropes due to hypotension following the insult.

**Table 3:** Hemodynamic support requirements.

	Sham	LPS	Hypoxia	LPS + Hypoxia	p value
Dopamine (µg/kg/min)	2.6 (1.9)	3.6 (2.1)	8.9 (8.3)	13.4 (6.0)	0.12
Dobutamine (µg/kg/min)	0 (0)	0 (0)	4.8 (7.0)	4.8 (4.8)	0.33
Noradrenaline (ng/kg/min)	0 (0)	0 (0)	34.9 (56.4)	18.6 (19.0)	0.44
Adrenaline (ng/kg/min)	0 (0)	0.9 (1.9)	147.9 (229.8)	169.6 (151.2)	0.30
10 mL/kg Saline Bolus (n)	0 (0)	0 (0)	0.5 (0.7)	0.4 (0.4)	0.10

Inotrope and saline bolus use (mean and standard deviation (SD)) during experimentation. There was no significant difference between all groups using ANOVA analysis. The Hypoxia and LPS + Hypoxia groups required higher doses of dopamine, dobutamine, adrenaline and saline than non-hypoxia groups (p ≤ 0.046). There was no effect of LPS on inotrope requirement.

### TUNEL

**Figure 1:**
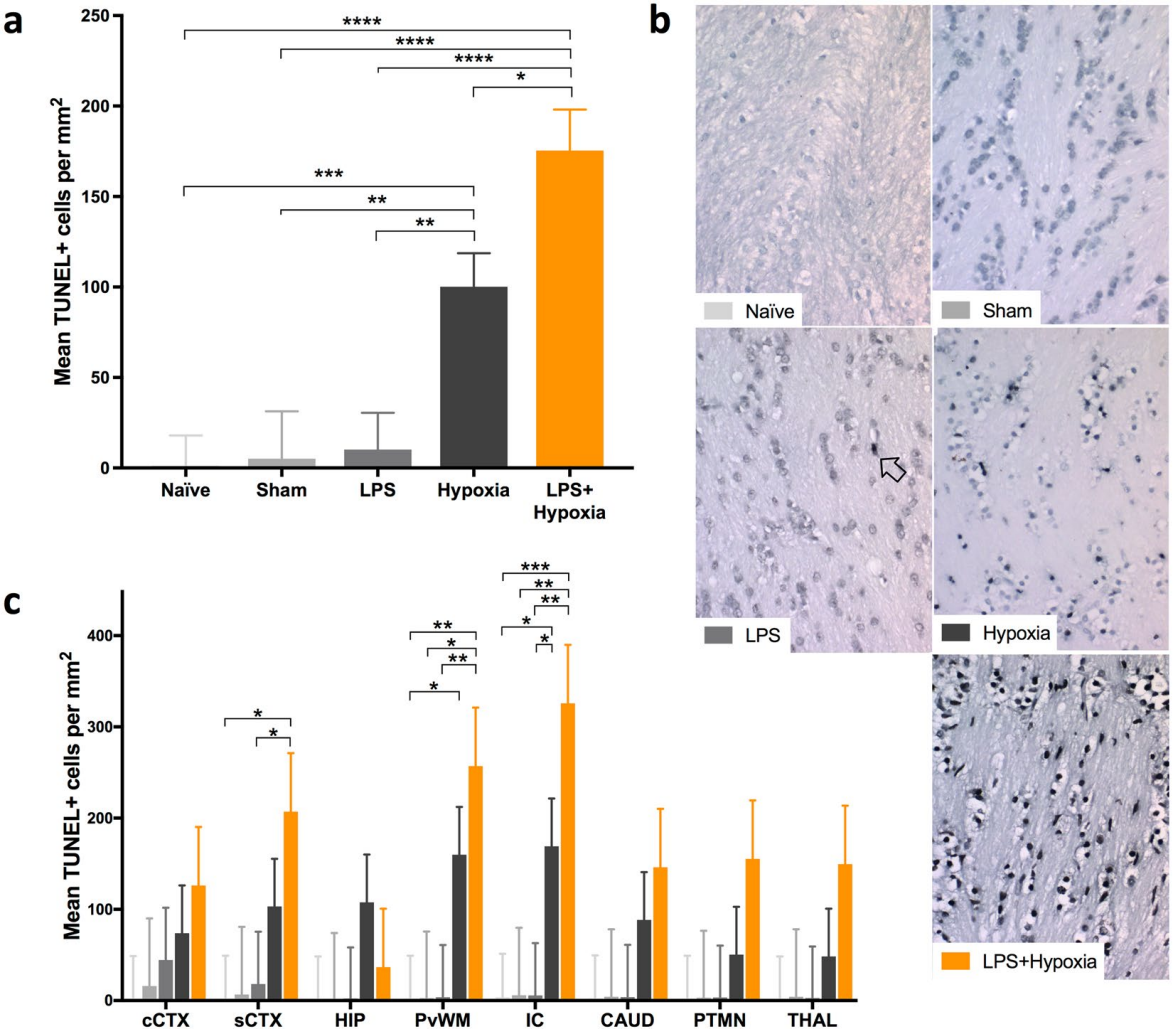
TUNEL histology. There was an overall increase in the estimated mean TUNEL+ cells per mm^2^ (pooled across region and R0/R1 levels) in the LPS + Hypoxia group versus all other groups (**a**). Representative sections are shown at x40 magnification from the internal capsule (**b**). A TUNEL+ cell is arrowed. On regional assessment, there was an increase in TUNEL+ cells for LPS + Hypoxia in the IC and PvWM versus Naïve, Sham and LPS, and in the sCTX versus Naïve and LPS (**c**). Cingulate cortex = cCTX; Sensorimotor cortex = sCTX; Hippocampus = HIP; Periventricular white matter = PvWM; Internal capsule = IC; Caudate = CAUD; Putamen = PTMN; Thalamus = THAL. Error bars represent standard error. ****p < 0.0001, ***p < 0.001, **p < 0.01, *p < 0.05.

Estimated mean TUNEL+ cell counts were similar for Naïve, Sham and LPS groups (Fig. 1a). The Hypoxia group TUNEL+ count was increased versus LPS, Sham and Naïve (p ≤ 0.004). LPS + Hypoxia had greater histological cell death than all other groups (p < 0.0001 versus Naïve, Sham, LPS and p = 0.011 versus Hypoxia). Representative photomicrographs from the internal capsule at 40x magnification are shown in Fig. 1b.

Regional comparisons are shown in Fig. 1c. LPS + Hypoxia had increased cell death in the internal capsule and periventricular white matter versus Naïve, Sham and LPS, and in the sensorimotor cortex versus Naïve and LPS (p ≤ 0.04). Hypoxia group had greater cell death in the internal capsule versus Naïve and LPS, and in the periventricular white matter versus Naïve (p ≤ 0.047).

### Cleaved Caspase-3

**Figure 2:**
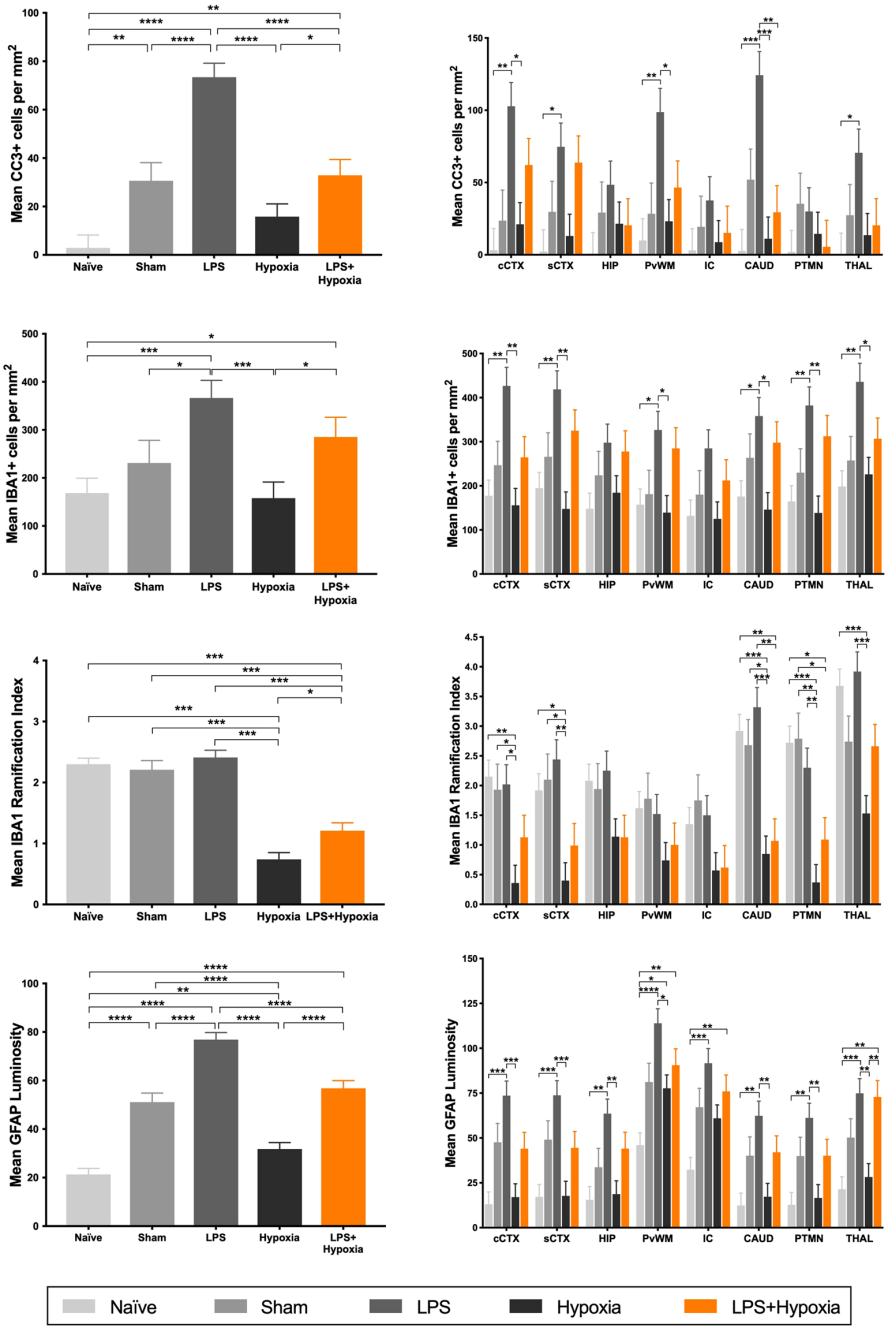
Estimated mean whole brain (left) and regional (right) cleaved caspase-3 (**a**), IBA1 cell count (**b**), IBA1 ramification index (**c**) and GFAP luminosity (**d**). CC3 positive cells markedly increased with LPS exposure. There was no effect of hypoxia in isolation on CC3. In combination, the increase in CC3 with LPS was attenuated by hypoxia exposure. Microglia number (IBA1 count) was increased by LPS exposure, whilst microglial activation (IBA1 ramification index) was increased by hypoxia exposure. Astrogliosis was increased from Naïve for all groups, maximal for LPS. Cingulate cortex = cCTX; Sensorimotor cortex = sCTX; Hippocampus = HIP; Periventricular white matter = PvWM; Internal capsule = IC; Caudate = CAUD; Putamen = PTMN; Thalamus = THAL. Error bars represent standard error. ****p < 0.0001, ***p < 0.001, **p < 0.01, *p < 0.05.

All groups, except Hypoxia, had a significant increase in CC3 positive cells compared with Naïve (Fig. 2a). CC3 staining was maximal for LPS, greater than all other groups (p ≤ 0.0001). The addition of hypoxia to LPS reduced CC3 count to be equivalent to Sham, but was still greater than Hypoxia alone (p = 0.044). On post hoc analysis, there was no correlation between CC3 positive and TUNEL+ cell count (r_s_ = 0.113).

### Microglia

**Figure 3:**
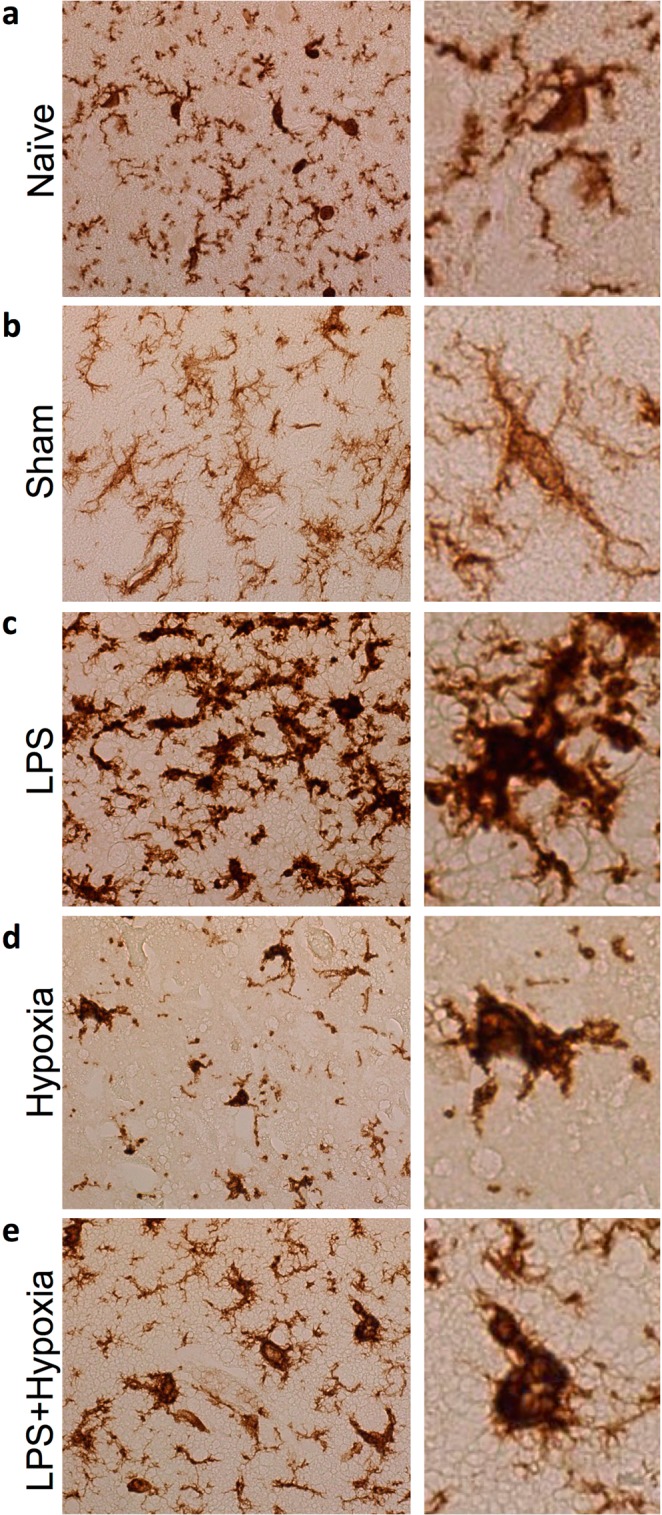
Representative IBA1 stained microglia in the cingulate cortex at x40 magnification, for Naïve (**a**), Sham (**b**), LPS (**c**), Hypoxia (**d**) and LPS + Hypoxia (**e**). In non-disease states, microglia have small cell bodies and multiple fine branches. LPS exposure subjectively increased branch thickness, while hypoxia induced amoeboid change.

Overall and regional brain IBA1 cell count (2b) and ramification index (2c) are shown in Fig. 2. Representative examples of microglia (IBA1 staining) are shown in Fig. 3. Microglia count was increased for LPS and LPS + Hypoxia, compared with Naïve and Hypoxia, and for LPS versus Sham (p ≤ 0.034). The microglial ramification index is a measure of microglial activation; activated microglia become amoeboid with fewer processes and therefore a lower ramification index. LPS alone did not alter ramification index compared to Sham and Naïve. Mean ramification index was lower for Hypoxia and LPS + Hypoxia compared with other groups (p < 0.0001). The Hypoxia group had a greater degree of microglial activation compared to the LPS + Hypoxia group (p = 0.007). These changes were consistent throughout the 8 brain regions.

### Astroglia

All groups had an increase in astrogliosis versus Naïve (p = 0.005 for Hypoxia, p < 0.0001 for all other groups) (Fig. 2d). Throughout all brain regions GFAP staining of reactive astrocytes was maximal for LPS, and was greater than each other group on whole brain analysis (p < 0.0001). The Hypoxia group GFAP luminosity was lower than Sham and LPS + Hypoxia (p < 0.0001).

An overview of all histological outcome variables is listed in Table 4.

**Table 4:** Histology summary.

	Sham	LPS	Hypoxia	LPS + Hypoxia
TUNEL+ cell death	—	—		
Caspase-3 cleavage			—	
Microglia count	—		—	
Microglial activation	—	—		
Astrogliosis				

An overview of histological outcome measures. A dash demonstrates no difference to Naïve. A single arrow represents an increase above Naïve. A double arrow represents an increase compared with the single arrow group, and a triple arrow an increase above the double arrow group.

### aEEG

**Figure 4:**
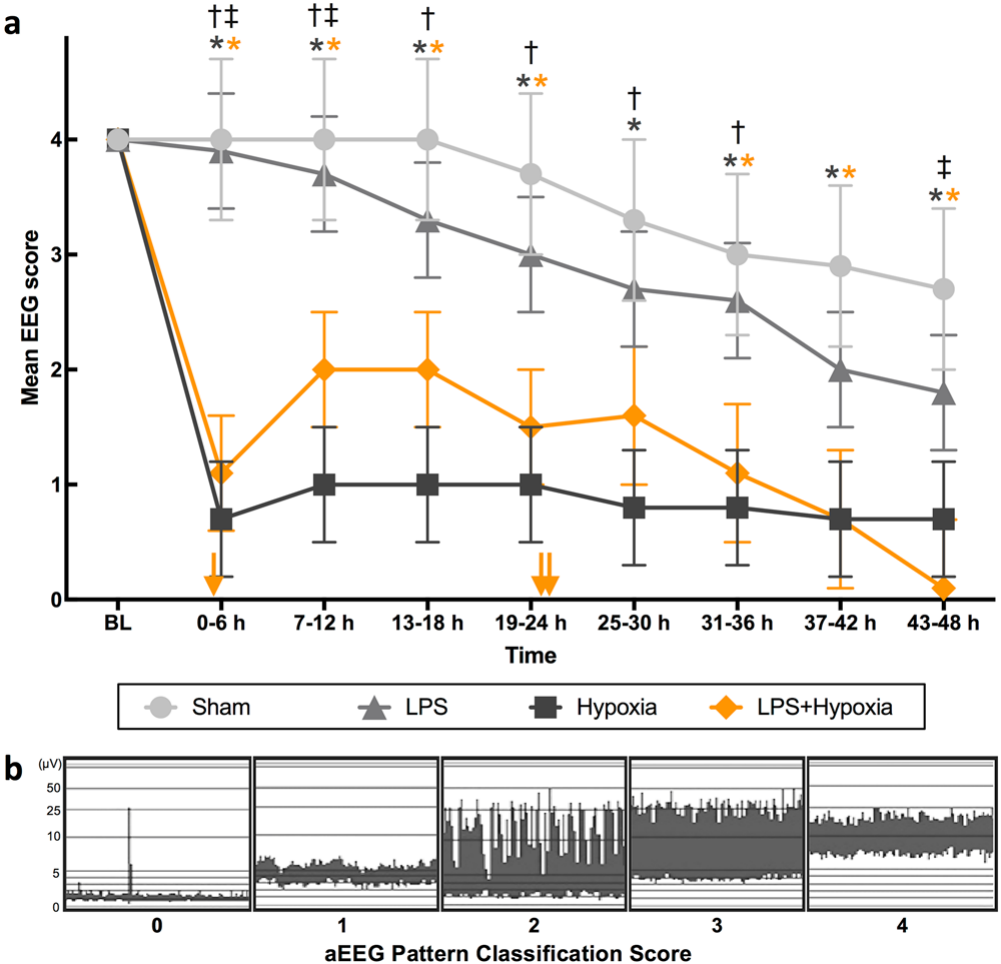
Mean aEEG score from baseline (BL) till 48 h, divided into 6 h time epochs (**a**), using pattern classification scoring system (**b**), where 0 = flat trace, 1 = low voltage, 2 = burst suppression, 3 = discontinuous normal voltage, and 4 = continuous normal voltage. After insult, Hypoxia group had lower scores than Sham at all time points (p ≤ 0.025), and than LPS until 36 h (p ≤ 0.021). LPS + Hypoxia had lower scores than Sham at all time points, except 25–30 h (p ≤ 0.038); and than LPS from 0–12 h, and again at 43–48 h (p ≤ 0.033). There was no difference between Hypoxia and LPS + Hypoxia. Phenobarbitone was given to one Hypoxia piglet at 6 h post insult (20 mg/kg), and one LPS + Hypoxia piglet at 22 h (20 mg/kg) and 39 h (10 mg/kg) post insult. Error bars represent standard error. *p < 0.05 vs. Sham (*colour denotes group), ^†^p < 0.05 LPS vs. Hypoxia, ^‡^p < 0.05 LPS vs. LPS + Hypoxia. Yellow arrows along the x-axis represent approximate time of death of the 3 LPS + Hypoxia subjects who died prematurely.

All piglets had a normal (score 4) aEEG at baseline (Fig. 4). Mean aEEG score for Hypoxia and LPS + Hypoxia was suppressed following insult, and remained suppressed throughout the experiment. There was no difference between mean aEEG score at any time point for Hypoxia compared to LPS + Hypoxia. The mean aEEG score was <4 in all groups by 52 h; there was a trend towards LPS aEEG score deteriorating earlier than Sham. Two Hypoxia piglets had electrographic seizures; one treated with 20 mg/kg phenobarbitone at 6 h post insult, and the other untreated at 8 and 12 h post insult. One LPS + Hypoxia piglet received 20 mg/kg phenobarbitone at 22 h post insult, followed by 10 mg/kg at 39 h.

### Haematology

**Figure 5:**
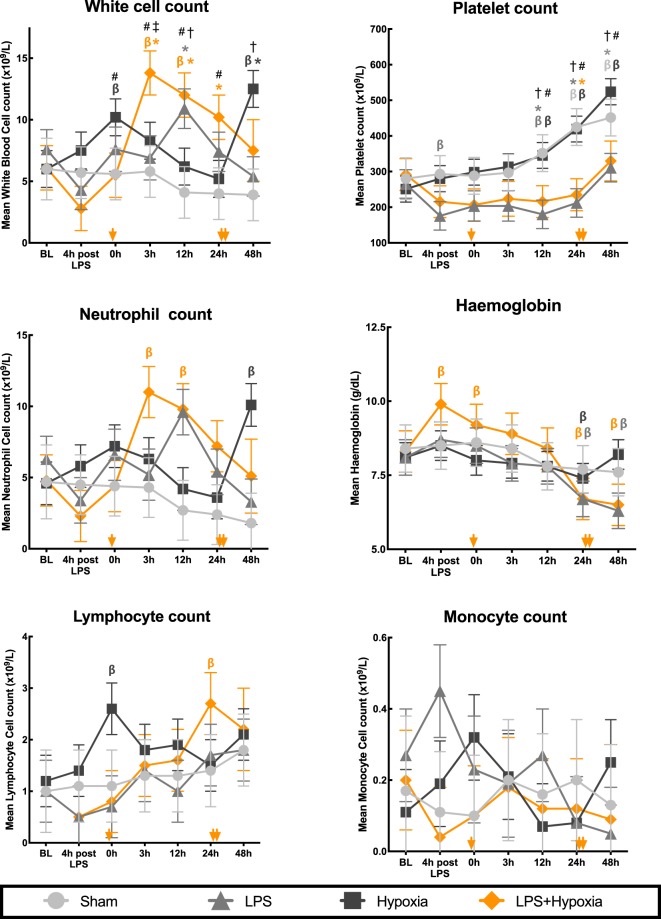
Change in haematology parameters throughout experimentation. WCC and platelet count analysis included between group comparisons at each time point. Error bars represent standard error. BL = baseline; β p < 0.05 change from BL (β colour denotes group), *p < 0.05 vs. Sham at same time point (*colour denotes group), ^†^p < 0.05 LPS vs. Hypoxia, ^‡^p < 0.05 LPS vs. LPS + Hypoxia, ^#^p < 0.05 Hypoxia vs. LPS + Hypoxia. Yellow arrows along the x-axis represent approximate time of death of the 3 LPS + Hypoxia subjects who died prematurely.

Serial haematology values are shown in Fig. 5. Total WCC, neutrophil, lymphocyte and monocyte counts were stable throughout the experiment in the Sham group. All other groups showed an increase in WCC from baseline. The LPS WCC (mainly neutrophils) peak occurred at 12 h (p ≤ 0.03 versus Sham and Hypoxia). LPS + Hypoxia had the highest and most sustained increase in WCC from 3 h to 24 h (p ≤ 0.036 compared to Sham and Hypoxia groups). There was a biphasic WCC increase from baseline for Hypoxia, with an immediate rise after the insult (p = 0.034), and a second peak at 48 h (p = 0.001), driven mainly by neutrophilia (p ≤ 0.002 versus Sham and LPS). Lymphocyte count increased for LPS + Hypoxia, peaking at 24 h (p = 0.005), later than the 3 h neutrophil peak. On post hoc analysis, peak WCC modestly correlated with TUNEL+ cell count (r = 0.57, p = 0.014) and with IBA1 ramification index (r = −0.64, p = 0.004). There was no correlation between peak WCC and any of GFAP (r_s_ = −0.26), IBA1 count (r = −0.40) or CC3 (r_s_ = −0.15).

Compared to baseline, Sham and Hypoxia developed a thrombocytosis, with a significant elevation from 24 h for Sham and from 12 h for Hypoxia (p ≤ 0.014). LPS infusion suppressed this rise in platelets: at all time points after baseline, the mean platelet counts for the LPS treated groups (LPS and LPS + Hypoxia) were lower than non-LPS groups (p ≤ 0.03). Following an initial increase, haemoglobin was lower than baseline in both LPS treated groups from 24 h onwards (p < 0.001).

### Spleen weight

There was no difference in spleen weight across all groups on ANOVA analysis. Spleen weight of those piglets treated with LPS (LPS and LPS + Hypoxia) suggests splenic hypertrophy when compared with non-LPS treated piglets (Sham and Hypoxia) −2.4 ± 0.4 g/kg body weight, versus 1.3 ± 0.2 g/kg body weight, (p = 0.006). There was no effect of hypoxia on spleen weight.

## Discussion

Bacterial LPS commenced 4 h prior to hypoxia and continued for 48 h in the newborn piglet resulted in an increase in mortality and an exacerbation of brain cell death compared to hypoxia alone. LPS alone did not increase cell death, but resulted in the greatest increases in microglial proliferation, reactive astrocytosis and cleavage of caspase-3. EEG recovery was suppressed by hypoxia, with no additional effect of LPS. LPS in both the non-hypoxia and hypoxia groups was associated with a suppression of platelet count. The spleen weight was greater in the LPS exposed groups, suggesting activation of the systemic immune response.

Our immunohistochemistry (TUNEL) findings are consistent with previous clinical studies which link antepartum inflammation with an increased risk of NE and brain injury following HI. For example, histological chorioamnionitis was found in 46% of placentae from one NE cohort, in comparison to 18% of healthy term infants from the same region^13^. Funisitis increases risk of NE in both high- (OR 9.29 (95% CI 1.36–63.06))^32^ and low- resource settings (OR 11.8 (95% CI 2.19–63.45))^15^. Neonatal bacteraemia has also been associated with an increased risk of NE^15,33^. Indeed, a number of observational studies suggest the risk of neurodevelopmental impairment after inflammation-sensitized HI is high^7,8,18,32^. The risk of developing CP is markedly increased with the combination of antenatal infection and a potentially birth-asphyxiating condition, compared to either in isolation (OR 78 (95% CI 4.8–406), versus OR 7.2 (95% CI 2.7–20) and 2.5 (95% CI 1.0–6.7) respectively)^18^. Similarly, chorioamnionitis in combination with MRI evidence of HI injury increases risk of CP (OR 17.5 (95% CI 3.3–93.4))^8^. In NE infants, raised pro-inflammatory cytokines at birth are associated with an increase in mortality and poor neurodevelopmental outcome^16,17,34^. In a large multicentre meta-analysis, mortality amongst infants with both Group B streptococcal sepsis and NE was higher than for infants with NE alone (21% compared with 13.7%, RR 2.07 (95% CI 1.47–2.91))^33^. We found a similarly increased mortality rate amongst our piglets with NE and inflammation.

The mechanism by which short-term preceding inflammation increases brain vulnerability to HI is complex. Pathways depend upon pathogen and timing of exposure. Gram-negative organisms and LPS act via TLR4 receptors, whilst gram positive organisms predominantly act via TLR2 receptors^35^. LPS, and other TLR agonists, increase the susceptibility to term HI in rodent models^19,21,24,36^. In models of LPS sensitized NE, TLR4 binding triggers many self-perpetuating pathways, including Myd88 dependent induction of nuclear factor kappa-light-chain-enhancer of activated B cells (NF-κB), microglial activation/proliferation and release of pro-inflammatory cytokines, including primary mediators of injury, interleukin 1 (IL-1) and Tumour necrosis factor alpha (TNFα)^5,19,22,24,37–41^. Activation of TLR4 by LPS before hypoxia is thought to suppress mitochondrial respiration, leading to a cumulative larger ATP depletion during hypoxia. Cells with decreased mitochondrial respiration are more vulnerable to glutamate excitotoxicity^42^. Additionally, LPS has been associated with in an increase in cyclooxygenase prostaglandin E_2_ pathway mediated brain injury and with early neutrophil infiltration^23,37,43^. Endogenous glucocorticoid stress response to LPS also contributes to neuronal injury^37,44^. Speculation following rodent studies^19^, that LPS increases brain injury by inducing hypotension prior to HI, is not supported by the results of this study; pre-hypoxia MABP was stable after 4 h of LPS, and higher in the LPS compared with saline treated piglets.

The timing of inflammation prior to an insult is important as shown in other animal models. In a rodent model, LPS 12 h prior to insult resulted in maximal tissue loss compared with earlier or later administration^24^. Eklind *et al*. demonstrated neuroprotection with LPS given 24 h prior to insult and injury exacerbation with LPS given 6 h or 72 h prior to insult in rodent pups^45^. Protective pre-conditioning with chronic LPS has been replicated in the rodent^46^ and preterm lamb^47,48^. The most frequently studied time interval between LPS and HI in the rodent has been 4h^19,20,24,41,44,49,50^. This time interval in the rodent is difficult to extrapolate to the clinical setting given the rodent’s accelerated life cycle. In this piglet study, we gave LPS 4 h prior to hypoxia, to validate the rodent studies. Only 4 h sensitization was modelled in our study.

Available evidence strongly supports that perinatal infection and inflammation can cause neurological stress and injury without a sentinel event. Neonates exposed to maternal infection are more likely to have low Apgar scores^7^, and those with sepsis demonstrate abnormal neurological symptoms in 63% of cases^51^. Amongst infants exhibiting NE, those without MRI evidence of HI injury have the greatest proportion of chorioamnionitis and the highest C-reactive protein levels^13^, suggesting encephalopathy due to infection and inflammation, rather than a sentinel event. On multivariate analysis, chorioamnionitis in isolation of birth asphyxia is independently associated with CP^7–9,18^. Surprisingly, in our study LPS only piglets had similar levels of TUNEL+ cell death to Naïve and Sham. This was despite evidence suggestive of a neuroinflammatory response, with the highest levels of reactive astrogliosis, microglial number and cleavage of caspase-3. These latter three processes may trigger a cascade of brain injury over a longer time period than the 52 h observed in our study^52^.

The dose of LPS used for this study was sufficient to cause a systemic response, as evidenced by a tachycardia in the two LPS groups at 4 h and increase in WCC. Temperature instability was not seen due to strict control by the Tecotherm mattress. There was a trend towards an earlier deterioration of the aEEG for LPS piglets compared with Sham. LPS, with and without hypoxia, resulted in an increase in circulating neutrophils and splenic hypertrophy, suggestive of activation of the adaptive immune system. In addition, LPS suppressed thrombocytosis; this may be related to platelet consumption and release of megakaryocyte inhibitors by activated platelets as occurs clinically in neonates with infection^53^.

Cleavage of caspase-3 was markedly increased in the LPS group, despite a low TUNEL+ count. Upregulation of CC3 by LPS without increased cell death has been reported^54,55^, and may be linked to alternative immune roles of caspases, including microglial and lymphocyte proliferation/function, cell differentiation and autophagy^56–58^. Astrogliosis, measured by GFAP, was also maximal in the LPS group and higher in LPS + Hypoxia than Hypoxia group; this may reflect the LPS-triggered TNFα, IL-1ß and IL-6 release, which are mediators of astrocyte proliferation and activation^38,59,60^.

Microglial cell counts were highest for LPS and LPS + Hypoxia groups, likely due to chemokine induced microglial proliferation by LPS. Unexpectedly, hypoxia alone did not increase microglial number^61,62^. Microglial activation state, considered important in quantifying the cell’s inflammatory response^5,22^, was most marked with Hypoxia alone followed by LPS + Hypoxia, the two groups with increased TUNEL+ cell death. The pronounced microglial activation with Hypoxia relative to LPS + Hypoxia may be related to a possible counteracting ‘hyper-ramification’ effect of LPS, previously demonstrated in adult mice^63^ or because there was insufficient time for microglial activation to evolve in the LPS + Hypoxia piglets who died prematurely^64^. The inverse relationship between Hypoxia and LPS + Hypoxia for cell death and microglial activation highlights the complex role that activated microglia contribute to both injury (an M1 pro-inflammatory phenotype) and neuroprotection and repair (an M2 anti-inflammatory restorative phenotype)^5,61,65,66^.

For both Hypoxia and LPS + Hypoxia, the increase in TUNEL+ cell death was without an increase in CC3. CC3 cell count was not correlated with TUNEL+ cell count. This suggests the observed TUNEL+ cell death occurred by processes independent of caspase 3, such as necrosis, necroptosis^57^, and non-caspase mediated apoptosis (for example, via the apoptosis inducing factor pathway)^67^. The Hypoxia group had reduced GFAP compared with Sham reflecting hypoxic astroglial injury. This reduction in GFAP/astroglial density at 48–72 h post HI has been demonstrated in the piglet previously, with subsequent recovery at 96h^68,69^. Hypoxic injury may also account for the reduction in GFAP, CC3 and microglial count seen for LPS + Hypoxia relative to LPS alone.

The Sham group demonstrated an increase in astrogliosis and CC3 in comparison with Naïve. The Sham and Naïve group differ in postnatal age at death, and in experimental intervention. GFAP expression increases with advancing maturity in the rodent^70^, pig^71^ and human^72^ brain, although it is unknown if 52 h maturation is sufficient justification for the incremental change seen here. We have previously demonstrated that surgery (tracheostomy) and brief anaesthesia (6 h isoflurane and fentanyl) is sufficient to mildly increase CC3 and TUNEL+ cells in the piglet^73^. Our current study was not powered to detect small differences in TUNEL+ count.

In this hypoxia model TUNEL+ staining was increased in the internal capsule, periventricular white matter and sensorimotor cortex, consistent with a watershed injury pattern seen in babies^74^ and primates^75^ typically following ‘prolonged partial asphyxia’; this injury pattern corresponds to specific motor^76^ and neurodevelopmental outcomes in children^77^. Our previous studies involved a hypoxic-ischemic insult induced by transient carotid artery occlusion; the pattern of injury was typically deep grey matter which is consistent with a sentinel event^26,28^. In comparison to previous studies using the carotid occlusion HI insult^26^, piglets were systemically more unstable, particularly in the LPS + Hypoxia group in which 3 out of 5 piglets died around 24 h. This reflects the systemic injury from global hypoxia and the contribution of LPS to systemic organ dysfunction.

There are some limitations to this study. This was a novel insult, without carotid occlusion or MRS cerebral metabolism monitoring^78^; this was aimed towards a more controlled insult and enabled continuous EEG. Despite new methodology and a lack of allocation concealment, we were able to maintain consistency in insult severity between the two hypoxia groups, as evidenced by similar insult parameters. There was a trend towards a shorter aEEG isoelectric recording in the LPS + Hypoxia group; this is likely to reflect the reduced tolerance to hypoxia in the LPS + Hypoxia group. Inotrope and phenobarbitone use, both potential mediating variables, were similar between the two hypoxia groups. The contribution of phenobarbitone to histological brain injury is unclear, with both neuroprotection and worsening of histological brain injury demonstrated following its use in preclinical neonatal models^79–81^. The Hypoxia group had a mildly elevated baseline pH of 7.55. The normal mean arterial pH for a piglet is 7.5^82^ so it is unlikely this had a meaningful biological effect. Other between-group differences in physiological parameters, excluding those immediately post insult, were within expected ranges and unlikely to affect outcome measures. Two piglets, one LPS and one Hypoxia, had brief respiratory arrests during experimentation. This may have contributed to in an increase in brain injury, which would result in an underestimation of the difference to the LPS + Hypoxia group.

The sample size is a study limitation. With this number of subjects we are only able to detect large differences in TUNEL+ cell death. Smaller but still potentially meaningful differences, for example between LPS and Sham/Naïve are not able to be determined. Given the small sample size, only male piglets were used to reduce intergroup variation. We were therefore not able to examine the increasingly recognized impact of sex on NE/HI outcome^83^. Inclusion of both sexes is a necessary area for future development of the model, and will require larger group sizes. The small sample size was compounded by the premature mortality in the LPS + Hypoxia group, resulting in heterogeneous duration of LPS exposure and time since hypoxia at histological specimen collection, and only *n* = 2 for EEG, physiology and haematology data after 24 h. We perceived the risk of ongoing mortality was high for this group, therefore further studies were considered unethical. Our future experiments using an LPS NE model, will change the insult protocol to use carotid occlusion which will reduce the burden of systemic hypoxia and will likely reduce animal losses.

We used bacterial LPS injection in this study and so modelled only gram-negative infection/endotoxaemia. Although gram-positive organisms are the most common cause of peripartum sepsis in high resource settings, gram-negative neonatal sepsis is increasing in frequency due to antibiotic prophylaxis^84^. In low- and middle-resource settings, gram-negative organisms, such as Klebsiella species and *E. coli*, surpass gram-positive organisms as the leading causative pathogens in neonatal sepsis^85,86^. Interestingly, the response to therapeutic hypothermia (HT) with sensitization from gram negative and positive organisms differs in rodent models. In a rodent HI model, prior LPS or PAM_3_CSK_4_ (a TLR2 agonist) caused a similar degree of brain injury^21^, but only the PAM_3_CSK_4_ sensitized rodents benefitted from HT^20,21^. A small observational study suggested HT for NE may be less protective with chorioamnionitis^87^. Indeed it is possible that HT may be harmful in the setting of infection^88–90^. In babies, HT causes chemokine-associated immunosuppression, with reduced peripheral leucocyte numbers, associated with a poorer long-term outcome if persisting after rewarming^91^. Furthermore, TH alters temporal cytokine profile^34^; reduces microglial activation^27^ and lowers blood pressure^92^, potentially contributing to injury and systemic instability in inflammation-sensitized NE. Large animal studies investigating the efficacy and safety of HT and other potential neuroprotective agents in inflammation-sensitized HI are required.

Our pre-clinical study gives a biological basis for the higher mortality and severe neurological outcome seen in babies with inflammation-sensitized NE. To optimize outcomes, it will be important to detect these babies, so that optimal therapies might be given. aEEG did not differentiate hypoxia from inflammation-sensitized hypoxia: both caused sustained aEEG depression. Suppression of thrombocytosis differentiated LPS exposure. WCC peak at 3 h discriminated LPS + Hypoxia from other groups, but the elevation was only moderate and still within the normal range; WCC peak was only modestly correlated with TUNEL+ cell death and microglial activation. Protein and gene expression show promise in delineating inflammation sensitization and may help to target neonatal therapies according to inflammation profile^93^.

We have shown in the newborn piglet undergoing intensive care support over 52 h, that LPS sensitization 4 h prior to hypoxia resulted in an increase in mortality and in overall brain cell death (TUNEL+ cells), mainly in the internal capsule, periventricular white matter and sensorimotor cortex. Our findings concur with data from rodent models; cell death following LPS-sensitized hypoxia was greater than combined cell death from hypoxia alone and LPS alone. In isolation, LPS did not increase cell death compared to naïve brain but LPS exposure alone resulted in the greatest increases in microglial proliferation, reactive astrogliosis and cleavage of caspase-3. EEG recovery was suppressed by hypoxia, with no additional effect of LPS. The evolution of white cell and platelet count throughout experimentation differentiated exposure to LPS and hypoxia with a suppression in thrombocytosis with LPS. Given the significant risk of adverse outcomes with co-existing inflammation and birth asphyxia, it will be important to determine the impact of HT and other neuroprotective interventions in this LPS-sensitized piglet model, to guide future clinical studies.

## Methods

The study was conducted according to UK Home Office Guidelines [Animals (Scientific procedures) Act, 1986] and complies with the ARRIVE guidelines. The study was approved by the Ethics Committee of UCL.

### Sample size calculation

Using previous piglet data, and 2 LPS pilots, we estimated that 5 piglets per intervention group were required to detect a difference in TUNEL+ cells of 45 cells/mm^2^, using a significance threshold of 5% and 80% power. Owing to lower anticipated variability, and to minimize animal use in accordance with ARRIVE guidelines, only 3 piglets were determined to be required in the Sham group.

### Animal experiments and surgical preparation

Male Large White piglets aged <36 h were included. Piglets were sedated with intramuscular midazolam (150 µg), and arterial O_2_ saturation monitored (Nonin Medical, MN, USA). The Naïve group (n = 7) was euthanized immediately following midazolam, within 10 minutes of arrival to the facility. Remaining piglets were anesthetized and surgically prepared. Isoflurane anaesthesia (3% v/v) was given to facilitate tracheostomy (Smiths Medical, Kent, UK) and was maintained (2–3%). Piglets were mechanically ventilated (SLE 2000, Surrey, UK) with PEEP 5, PIP 20 and inspiratory time 0.5 seconds; Fraction of inspired oxygen (FiO2) and respiratory rate were adjusted to maintain partial pressure of oxygen (PaO_2_) and carbon dioxide (PaCO_2_) at 8–13 kPa and 4.5–6.5 kPa, respectively.

A 4 French double-lumen umbilical venous catheter (Vygon, Swindon, UK) was inserted for infusion of maintenance fluids (10% dextrose, 60 ml/kg/day, reduced to 40 ml/kg/day post insult), fentanyl (3 μg/kg/h) and antibiotics (benzylpenicillin 50 mg/kg every 12 h and gentamicin 4 mg/kg every 24 h). A 2 French central venous catheter (Vygon) was sited in the axillary or brachial vein for infusion of LPS or saline. A 2.5 French umbilical arterial catheter (Vygon) was inserted for continuous monitoring of heart rate and arterial blood pressure (MABP), and intermittent blood sampling (Abbot Laboratories, UK). Arterial lines were maintained by infusing 0.9% saline solution (0.3 mL/h) with 1 IU/mL heparin.

Duration of surgery was 1–2 h, after which piglets were positioned prone. A post–surgery blood gas was taken to ensure no intraoperative hypoxia or hypoperfusion. All piglets received continuous physiological monitoring (SA instruments) and intensive life support throughout the study. Rectal temperature was maintained in the normothermic range (38.0–39.0 °C) for all piglets for the duration of the experiment, using a radiant warmer during surgery and subsequently a heated water mattress (Tecotherm). Infusions of crystalloid (0.9% saline, 10 mL/kg bolus, prn, maximum once daily) and inotropes (escalation protocol using dopamine 5–25 μg/kg/min, dobutamine 5–20 μg/kg/min, noradrenaline 0.1–1 μg/kg/min, and adrenaline 0.1–1 μg/kg/min) maintained MABP >35 mmHg. Hyperkalaemia, hyperglycaemia and metabolic acidosis were managed in accordance with clinical neonatal guidelines.

### Experimental groups

**Figure 6:**
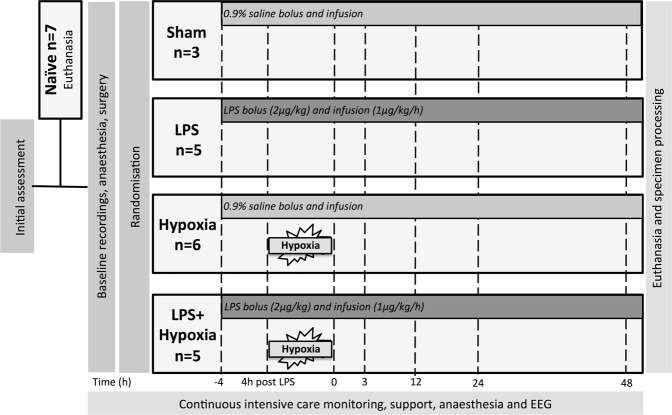
Study time-line. Following initial weight and wellbeing assessment, 7 piglets were randomly assigned to immediate euthanasia (Naïve). Following baseline recordings and tracheostomy, remaining piglets were randomized to receive (i) Saline bolus and infusion alone (Sham, n = 3), (ii) LPS bolus and infusion alone (LPS, n = 5), (iv) hypoxia 4 h after saline bolus (Hypoxia, n = 6) and (v) hypoxia 4 h after LPS bolus (LPS + Hypoxia, n = 5). Blood samples were collected at baseline, 4 h after bolus, end of hypoxia (time 0), and at 3, 12, 24 and 48 h after time 0. Piglets were maintained under meticulous intensive care for 48 h following hypoxia, prior to euthanasia and histological specimen collection. EEG was acquired continuously throughout.

Approximately 1 h following surgery and baseline recordings, piglets were randomized by computer generated sequence to (i) Sham (n = 3); (ii) LPS (LPS, n = 5); (iii) Hypoxia (Hypoxia, n = 6); and (iv) LPS and Hypoxia (LPS + Hypoxia, n = 5) (Fig. 6). LPS and LPS + Hypoxia piglets were given a bolus of 2 μg/kg LPS (*Escherichia coli* (*E. coli*), Sigma LPS O55:B5) followed by a continuous infusion of 1 μg/kg/h LPS for the duration of the experiment. Sham and Hypoxia piglets had a matched bolus of 0.9% saline (0.5 ml/kg), followed by a continuous saline infusion (0.25 ml/kg/h).

### Hypoxic insult

After the 4 h infusion, Hypoxia and LPS + Hypoxia piglets underwent a global hypoxia insult. FiO_2_ was reduced in a stepwise fashion to 4% over 3 minutes and held for 10 minutes. This was followed by a further 10 to 30 minutes of FiO_2_ 6–12% titrated to standardized parameters, including MABP 26–30 mmHg, and sustained isoelectric EEG (<5 uV). The insult was terminated if cardiac arrest was imminent indicated by profound hypotension or bradycardia.

### EEG acquisition

Multichannel six-lead EEG (Nicolet, Care Fusion, Wisconsin, USA) was acquired at baseline and continued throughout. aEEG recordings were classified hourly according to the pattern classification^94^ by 2 experienced clinical assessors (KM and IL), blind to treatment allocation. A score of 0 was given for flat trace; 1 continuous low voltage; 2 burst suppression; 3 discontinuous normal voltage and 4 continuous normal voltage. Each piglet’s scores were averaged over 6 h periods. Seizure activity was quantified. Electrographic seizures were treated with Phenobarbitone, in accordance with clinical neonatal guidelines.

### Haematology

Arterial blood samples were collected at baseline, 4 h after LPS/saline bolus, at end of hypoxic insult (or equivalent time for non-hypoxia piglets), and at 3, 12, 24 and 48 h post insult time. Samples were stored at 4 °C for a maximum of 72 h, and underwent external blinded complete blood film examination (Royal Veterinary College, Hawkshead, UK). Analyses included total white cell (WCC), neutrophil, lymphocyte, monocyte and platelet count and haemoglobin.

### Brain Histology

52 h after bolus (48 h post hypoxic insult for Hypoxia and LPS + Hypoxia groups), piglets were euthanized using pentobarbital (1 g/kg). Organs were fixed through a transcardial perfusion with cold phosphate-buffered saline, followed by 4% phosphate-buffered paraformaldehyde. The spleen was removed and weighed prior to paraformaldehyde perfusion. The brain was dissected out and post fixed at 4 °C in 4% paraformaldehyde for 7 days. Coronal slices (5 mm thick) of the right hemisphere were embedded in paraffin wax and sectioned (5 µm thick). For each animal, 2 sections, one taken through the hippocampus (R1), and another 5 mm anterior (R0) were assessed for each stain.

To quantify histological cell death, sections were stained for nuclear DNA fragmentation using histochemistry with Terminal deoxynucleotidyl transferase dUTP nick end labelling (TUNEL). In addition, apoptosis was assessed by Cleaved Caspase-3 (CC3) immunoreactivity. Glial activation was quantified using astrocyte Glial Fibrillary Acidic Protein (GFAP) and microglial ionized calcium-binding adaptor molecule (IBA1) immunoreactivity.

For all histochemical and immunohistochemical stains, brain sections were dehydrated in xylene (3 × 10 min) and rehydrated in graded ethanol solutions (100–70%), followed by double-distilled water. For TUNEL, the sections were pre-treated for 15 min in 3% H_2_O_2_ in methanol to remove endogenous peroxidase, followed by a 15-min peptidase pre-digestion with 20 µg/mL proteinase K (Promega) at 65 °C, and then incubated at 37 °C for 2 h with the TUNEL solution (Roche) containing biotinylated dUTP. For CC3 and IBA1, pre-treatment was performed with Ventana CC1 (950-124), equivalent to EDTA buffer, for 32 min. For GFAP, Protease 1 (0.38 mg/mL alkaline protease enzyme activity), for 4 min was used. Incubation with primary rabbit antibody was performed against CC3 (1:100) (Cell Signalling 9661 L) for 32 min, IBA1 (1:250) (WAKO 019-19741) for 4 h or GFAP (1:1000) (DAKO Z0334) for 32 min. Incubation with a secondary swine anti-rabbit immunoglobulin (DAKO E0343) was performed for either 44 min (CC3), 1 h (IBA1) or 32 min (GFAP).

The biotin residues were detected with the avidin-biotinylated horseradish peroxidase complex (ABC, Vector Laboratories) and visualized with diaminobenzidine/H_2_O_2_ (Sigma), with CoCl_2_ and NiCl_2_ included to intensify TUNEL histochemistry. The sections were dehydrated in graded alcohol and xylene and mounted with Depex (VWR), or alternatively, mounted with Vectashield + 4′,6-diamidino-2-phenylindole (DAPI) aqueous mounting media (Vector Labs), to facilitate total cell number counts during analysis of IBA1 and CC3.

Investigators blind to the treatment group performed analyses in 8 brain regions (cingulate cortex, sensorimotor cortex, hippocampus, periventricular white matter, internal capsule, caudate nucleus, putamen and thalamus). For each section and brain region, TUNEL+ nuclei were counted in three fields (at x40 magnification, with an area of 0.066 mm^2^) and the average converted into counts per mm^2^.

IBA1 positive cell body count was similarly performed. In addition, IBA1 positive microglial cell bodies and branch density were calculated at x40 magnification using a 0.049 mm × 0.049 mm square grid, placed in three fields for all brain regions and counting the number of cell bodies within the grid (C) and the average number of branches crossing the 3 horizontal and 3 vertical 0.49 mm gridlines (B). The microglial ramification index was calculated as (B^2^/C).

CC3 immunoreactive cells were counted in three fields (at ×20 magnification with an area of 0.164mm^2^) and the average converted into counts per mm^2^.

To quantify GFAP immunoreactivity optical luminosity values were calculated by deducting mean brightness values of the tissue (three fields per region at x20 magnification) from the mean brightness of the blank region of the corresponding slide^95^.

### Statistical methods

Using SAS JMP® Pro v14.0.0, analysis of variance models were fitted to give estimates of expected treatment group mean values and difference between means for TUNEL (primary outcome measure), secondary histology parameters (IBA1, GFAP and CC3), EEG, WCC and platelet count, and also a comparison to baseline was calculated for haematology values. Least Squares Means models were used to account for the imbalance in treatment group sizes overall and within measures. For TUNEL and other histology measurements, the effects of Treatment, Region and the interaction between them were fitted to the mean result for each subject averaged across replicate measurements within each region. For EEG and haematology, a model with terms for Treatment, Time Interval (as a factor) and the interaction between them, and a random subject effect to take into account the repeated measures structure was fitted to the results for each subject averaged across each time interval. Least square means are shown graphically with standard error bars. Using GraphPad Prism® v7 software, Tukey-Kramer multiple comparisons correction was applied for between group regional comparisons of secondary histology outcome parameters. Post hoc Pearson’s (r) or Spearman’s (r_s_) correlation between histological and haematology parameters were performed using IBM SPSS® v22 software. Physiological data were analysed with IBM SPSS® v22 software, using ANOVA and Tukey-Kramer multiple comparison correction for group comparisons and T test or Mann Whitney U test as appropriate for pairwise comparisons.

## Data Availability

The datasets used and/or analysed during the current study are available from the corresponding author on reasonable request
